# Supplementary material for: Identification of Genes Whose Expression Overlaps Age Boundaries and Correlates with Risk Groups in Paediatric and Adult Acute Myeloid Leukaemia
Source: Cancers (Basel). 2020 Sep 27;12(10):2769. doi: 10.3390/cancers12102769 (PMC7650662; doi:10.3390/cancers12102769)
Supplement: Supplementary file 1 [file cancers-12-02769-s001.zip › cancers-940887 proofed Figure S1.docx]

**Supplementary Materials:**

Identification of Genes Whose Expression Overlaps Age Boundaries and Correlates with Risk Groups in Paediatric and Adult Acute Myeloid Leukaemia

Lindsay Davis, Ken I. Mills, Kim H. Orchard and Barbara-Ann Guinn


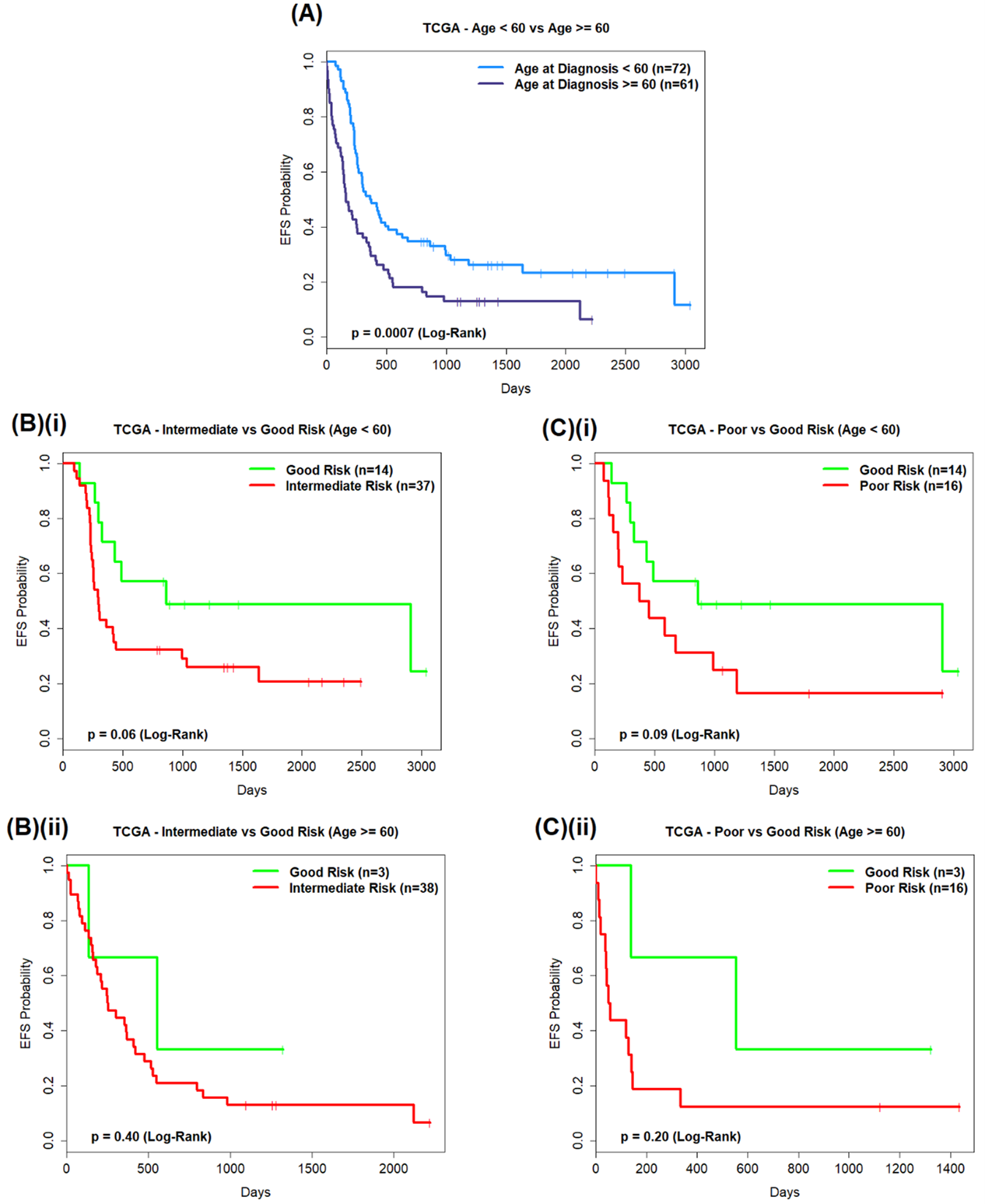


**Figure S1:** Pairwise comparisons of event-free survival (EFS) of total (**A**) TCGA patients by age < 60 and ≥ 60 years (*p* = 0.0007), and pairwise comparisons of TCGA patients by (**B**) intermediate versus good risk for those (**i**) < 60 years and (**ii**) ≥ 60 years of age (*p* = 0.06 and 0.40, respectively), and by (**C**) poor versus good risk for those (**i**) < 60 years and (**ii**) ≥ 60 years of age (*p* = 0.09 and 0.20, respectively).
