# Supplementary material for: Identification of Genes Whose Expression Overlaps Age Boundaries and Correlates with Risk Groups in Paediatric and Adult Acute Myeloid Leukaemia
Source: Cancers (Basel). 2020 Sep 27;12(10):2769. doi: 10.3390/cancers12102769 (PMC7650662; doi:10.3390/cancers12102769)
Supplement: Supplementary file 1 [file cancers-12-02769-s001.zip › cancers-940887 Table S2 - Pathway analysis all significant DEGs of interest FINAL 270520 (1).docx]

|  | **Supplementary Table 2**  **(A) All Significant DEGs of Interest** | | |  | |  | |  | |  | | | |
| --- | --- | --- | --- | --- | --- | --- | --- | --- | --- | --- | --- | --- | --- |
|  | **Patient Subgroup Comparison** | | **KEGG (2016) Term** | **KEGG (2016) Overlap** | | **KEGG (2016) Combined Score** | | **Overlapping KEGG (2016) Genes** | |  | | | |
| **TARGET** | Standard Risk vs Low Risk | | Mineral absorption_Homo sapiens_hsa04978 | 2/51 | | 10.73 | | SLC34A2;STEAP1 | |  | | | |
|  |  |  | Legionellosis_Homo sapiens_hsa05134 | 2/55 | | 10.62 | | CASP1;TLR4 | |  | | | |
|  |  |  | Fanconi anemia pathway_Homo sapiens_hsa03460 | 2/53 | | 10.28 | | FANCC;BRCA2 | |  | | | |
|  |  |  | Pertussis_Homo sapiens_hsa05133 | 2/75 | | 8.50 | | CASP1;TLR4 | |  | | | |
|  |  |  | Salmonella infection_Homo sapiens_hsa05132 | 2/86 | | 7.89 | | CASP1; TLR4 | |  | | | |
|  |  |  | Pathways in cancer_Homo sapiens_hsa05200 | 3/397 | | 7.51 | | DAPK1; BIRC5; BRCA2 | |  | | | |
|  |  |  | Hepatitis B_Homo sapiens_hsa05161 | 2/146 | | 7.06 | | BIRC5; TLR4 | |  | | | |
|  |  |  | Influenza A_Homo sapiens_hsa05164 | 2/175 | | 6.38 | | CASP1; TLR4 | |  | | | |
|  |  |  | Vitamin digestion and absorption_Homo sapiens_hsa04977 | 1/24 | | 3.99 | | FOLH1 | |  | | | |
|  |  | | Bladder cancer_Homo sapiens_hsa05219 | 1/41 | | 3.81 | | DAPK1 | |  | | | |
| **TCGA** | Poor Risk vs Good Risk | | Mineral absorption_Homo sapiens_hsa04978 | 2/51 | | 13.79 | | SLC34A2; STEAP1 | |  | | | |
|  |  |  | Vascular smooth muscle contraction_Homo sapiens_hsa04270 | 1/120 | | 4.77 | | MYH11 | |  | | | |
|  |  |  | Tight junction_Homo sapiens_hsa04530 | 1/139 | | 4.03 | | MYH11 | |  | | | |
|  | Intermediate Risk vs Good Risk | | Mineral absorption_Homo sapiens_hsa04978 | 2/51 | | 13.79 | | SLC34A2; STEAP1 | |  | | | |
|  |  |  | Vitamin digestion and absorption_Homo sapiens_hsa04977 | 1/24 | | 7.58 | | FOLH1 | |  | | | |
|  |  |  | Alanine, aspartate and glutamate metabolism_Homo sapiens_hsa00250 | 1/35 | | 6.37 | | FOLH1 | |  | | | |
|  |  |  | Adherens junction_Homo sapiens_hsa04520 | 1/74 | | 5.41 | | SSX2IP | |  | | | |
|  |  |  | Vascular smooth muscle contraction_Homo sapiens_hsa04270 | 1/120 | | 4.56 | | MYH11 | |  | | | |
|  |  |  | Tight junction_Homo sapiens_hsa04530 | 1/139 | | 3.86 | | MYH11 | |  | | | |
|  |  |  | Metabolic pathways_Homo sapiens_hsa01100 | 1/1239 | | 1.06 | | FOLH1 | |  | | | |
|  |  | |  |  | |  | |  | |  | | | |
|  |  | |  |  | |  | |  | |  | | | |
|  | | **(B) Downregulated (log2FC < 0) Significant DEGs of Interest Only** | |  | | |  | | | | |  |  |
|  | | **Patient Subgroup Comparison** | **KEGG (2016) Term** | **KEGG (2016) Overlap** | **KEGG (2016) Combined Score** | | **Overlapping KEGG (2016) Genes** | |  | |  |  |  |
| **TARGET** | | Standard Risk vs Low Risk | Vitamin digestion and absorption_Homo sapiens_hsa04977 | 1/24 | 8.27 | | FOLH1 | |  | |  |  |  |
|  |  |  | Non-small cell lung cancer_Homo sapiens_hsa05223 | 1/56 | 6.94 | | FHIT | |  | |  |  |  |
|  |  |  | Bladder cancer_Homo sapiens_hsa05219 | 1/41 | 6.91 | | DAPK1 | |  | |  |  |  |
|  |  |  | Alanine, aspartate and glutamate metabolism_Homo sapiens_hsa00250 | 1/35 | 6.90 | | FOLH1 | |  | |  |  |  |
|  |  |  | Small cell lung cancer_Homo sapiens_hsa05222 | 1/86 | 5.41 | | FHIT | |  | |  |  |  |
|  |  |  | Purine metabolism_Homo sapiens_hsa00230 | 1/176 | 3.94 | | FHIT | |  | |  |  |  |
|  |  |  | MicroRNAs in cancer_Homo sapiens_hsa05206 | 1/297 | 3.33 | | BCL2L2 | |  | |  |  |  |
|  |  |  | Pathways in cancer_Homo sapiens_hsa05200 | 1/397 | 3.11 | | DAPK1 | |  | |  |  |  |
| **TCGA** | | Poor vs Good Risk | Vascular smooth muscle contraction_Homo sapiens_hsa04270 | 1/120 | 9.57 | | MYH11 | |  | |  |  |  |
|  |  |  | Tight junction_Homo sapiens_hsa04530 | 1/139 | 8.31 | | MYH11 | |  | |  |  |  |
|  |  | Intermediate vs Good Risk | Vitamin digestion and absorption_Homo sapiens_hsa04977 | 1/24 | 10.19 | | FOLH1 | |  | |  |  |  |
|  |  |  | Alanine, aspartate and glutamate metabolism_Homo sapiens_hsa00250 | 1/35 | 8.65 | | FOLH1 | |  | |  |  |  |
|  |  |  | Vascular smooth muscle contraction_Homo sapiens_hsa04270 | 1/120 | 6.79 | | MYH11 | |  | |  |  |  |
|  |  |  | Tight junction_Homo sapiens_hsa04530 | 1/139 | 5.84 | | MYH11 | |  | |  |  |  |
|  |  |  | Metabolic pathways_Homo sapiens_hsa01100 | 1/1239 | 2.85 | | FOLH1 | |  | |  |  |  |

|  |  |  |  |  |  |  |  |  |
| --- | --- | --- | --- | --- | --- | --- | --- | --- |
|  |  |  |  |  |  |  |  |  |
|  | **(C) Upregulated (log2FC > 0) Significant DEGs of Interest Only** | |  | |  | | |  |
|  | **Patient Subgroup Comparison** | **KEGG (2016) Term** | **KEGG (2016) Overlap** | **KEGG (2016) Combined Score** | **Overlapping KEGG (2016) Genes** |  |  |  |
| **TARGET** | Standard Risk vs Low Risk | Mineral absorption_Homo sapiens_hsa04978 | 2/51 | 12.39 | SLC34A2;STEAP1 |  |  |  |
|  |  | Legionellosis_Homo sapiens_hsa05134 | 2/55 | 12.30 | CASP1;TLR4 |  |  |  |
|  |  | Fanconi anemia pathway_Homo sapiens_hsa03460 | 2/53 | 11.88 | FANCC;BRCA2 |  |  |  |
|  |  | Pertussis_Homo sapiens_hsa05133 | 2/75 | 9.98 | CASP1;TLR4 |  |  |  |
|  |  | Salmonella infection_Homo sapiens_hsa05132 | 2/86 | 9.34 | CASP1;TLR4 |  |  |  |
|  |  | Hepatitis B_Homo sapiens_hsa05161 | 2/146 | 8.66 | BIRC5;TLR4 |  |  |  |
|  |  | Influenza A_Homo sapiens_hsa05164 | 2/175 | 8.05 | CASP1;TLR4 |  |  |  |
|  |  | Pathways in cancer_Homo sapiens_hsa05200 | 2/397 | 5.23 | BIRC5;BRCA2 |  |  |  |
|  |  | Malaria_Homo sapiens_hsa05144 | 1/49 | 4.91 | TLR4 |  |  |  |
|  |  | Amyotrophic lateral sclerosis (ALS)_Homo sapiens_hsa05014 | 1/51 | 4.46 | CASP1 |  |  |  |
| **TCGA** | Poor Risk vs Good Risk | Mineral absorption_Homo sapiens_hsa04978 | 2/51 | 14.09 | SLC34A2;STEAP1 |  |  |  |
|  | Intermediate Risk vs Good Risk | Mineral absorption_Homo sapiens_hsa04978 | 2/51 | 15.18 | SLC34A2;STEAP1 |  |  |  |
|  |  | Adherens junction_Homo sapiens_hsa04520 | 1/74 | 6.28 | SSX2IP |  |  |  |
|  |  |  |  |  |  |  |  |  |
